# Factor Structure, Reliability and Measurement Invariance of the Alberta Context Tool and the Conceptual Research Utilization Scale, for German Residential Long Term Care

**DOI:** 10.3389/fpsyg.2016.01339

**Published:** 2016-09-07

**Authors:** Matthias Hoben, Carole A. Estabrooks, Janet E. Squires, Johann Behrens

**Affiliations:** ^1^Knowledge Utilization Studies Program, Faculty of Nursing, University of AlbertaEdmonton, AB, Canada; ^2^Medical Faculty, Institute of Health and Nursing Sciences, Martin Luther University of Halle-WittenbergHalle, Germany; ^3^Network Aging Research, Heidelberg UniversityHeidelberg, Germany; ^4^Faculty of Health Sciences, School of Nursing, University of OttawaOttawa, ON, Canada; ^5^Clinical Epidemiology Program, Ottawa Hospital Research Institute, Ottawa HospitalOttawa, ON, Canada

**Keywords:** Alberta Context Tool, conceptual research utilization scale, organizational context, best practice use, psychometric testing, confirmatory factor analysis, measurement invariance, residential long term care

## Abstract

We translated the Canadian residential long term care versions of the Alberta Context Tool (ACT) and the Conceptual Research Utilization (CRU) Scale into German, to study the association between organizational context factors and research utilization in German nursing homes. The rigorous translation process was based on best practice guidelines for tool translation, and we previously published methods and results of this process in two papers. Both instruments are self-report questionnaires used with care providers working in nursing homes. The aim of this study was to assess the factor structure, reliability, and measurement invariance (MI) between care provider groups responding to these instruments. In a stratified random sample of 38 nursing homes in one German region (Metropolregion Rhein-Neckar), we collected questionnaires from 273 care aides, 196 regulated nurses, 152 allied health providers, 6 quality improvement specialists, 129 clinical leaders, and 65 nursing students. The factor structure was assessed using confirmatory factor models. The first model included all 10 ACT concepts. We also decided a priori to run two separate models for the scale-based and the count-based ACT concepts as suggested by the instrument developers. The fourth model included the five CRU Scale items. Reliability scores were calculated based on the parameters of the best-fitting factor models. Multiple-group confirmatory factor models were used to assess MI between provider groups. Rather than the hypothesized ten-factor structure of the ACT, confirmatory factor models suggested 13 factors. The one-factor solution of the CRU Scale was confirmed. The reliability was acceptable (>0.7 in the entire sample and in all provider groups) for 10 of 13 ACT concepts, and high (0.90–0.96) for the CRU Scale. We could demonstrate partial strong MI for both ACT models and partial strict MI for the CRU Scale. Our results suggest that the scores of the German ACT and the CRU Scale for nursing homes are acceptably reliable and valid. However, as the ACT lacked strict MI, observed variables (or scale scores based on them) cannot be compared between provider groups. Rather, group comparisons should be based on latent variable models, which consider the different residual variances of each group.

## Introduction

Use of best practices based on research (research utilization) is less than optimal in German residential long term care (LTC) settings (Kuske et al., 2009; Meyer et al., 2009; Majic et al., 2010; Treusch et al., 2010; Wilborn and Dassen, 2010; Reuther et al., 2013) with far-reaching consequences for the highly vulnerable residents in LTC. In 2011, the 12,354 LTC facilities in Germany were home to 743,120 residents (Statistisches Bundesamt, 2013). These LTC residents each typically suffer from four to five medical conditions (Balzer et al., 2013), such as: dementia diagnosis (50–70% of the residents), bladder or bowel incontinence (70–80%), oral-dental problems (50–90%), and mood or behavior problems (25–50%). Furthermore, many are functionally impaired (e.g., hearing problems, visual limitations, decreased communication abilities, reduced mobility) and are highly vulnerable to infections, falls and fractures, malnutrition, pressure ulcers, and many other risks (Lahmann et al., 2010; Volkert et al., 2011; Balzer et al., 2013). Due to these complex care needs LTC residents are particularly vulnerable to problems in quality of care, and it is important that caregivers are providing care in line with research based best practices.

Organizational context factors are thought to be centrally important to implementing research findings for improved quality of care (Greenhalgh et al., 2004; Dopson and Fitzgerald, 2005; Meijers et al., 2006; Squires et al., 2013a). For example, in their systematic review Kaplan et al. (2010) found that important factors for the success of quality improvement initiatives are leadership from top management, organizational culture, data infrastructure and information systems, years involved in quality improvement initiatives, physician involvement, microsystem motivation to change, resources, and team leadership. However, Kaplan et al. found key limitations in the literature: (a) an insufficient theoretical foundation, (b) insufficiently defined contextual factors, and (c) lack of well-specified measures. Especially in LTC settings, contextual factors are not well understood and their measurement has rarely been addressed (Estabrooks et al., 2009a; Masso and McCarthy, 2009; Boström et al., 2012; Rahman et al., 2012). This is particularly true for German LTC where dissemination and implementation research has played a small role and few such research tools are available (Hoben et al., 2014b). In three previous publications (Hoben et al., 2013, 2014a,b) we pointed out the lack of research tools to study the association of organizational context factors (e.g., leadership, organizational culture, feedback) with research utilization in German LTC settings.

One of the most pressing gaps in dissemination and implementation (DI) research is the lack of well-developed outcomes and measurement tools (Graham et al., 2010; Proctor et al., 2011; Proctor and Brownson, 2012). By dissemination we mean “an active approach of spreading evidence-based interventions to the target audience via determined channels using planned strategies” (Rabin and Brownson, 2012, p. 26). Implementation “is the process of putting to use or integrating evidence-based interventions within a setting” (Rabin and Brownson, 2012, p. 26). Reliable and valid research tools equip researchers to test theoretical assumptions about dissemination and implementation processes, and about the effectiveness of strategies, by using statistical methods in large samples (Proctor and Brownson, 2012). A lack of robust research tools hampers our understanding of how these processes work and how they can be effectively improved (Proctor and Brownson, 2012).

With the LTC versions of the Alberta Context Tool (ACT) (Estabrooks et al., 2011b) and the Conceptual Research Utilization (CRU) Scale (Squires et al., 2011a), we have two robust and widely used tools developed in Canada with which to study organizational context and research utilization in LTC settings. The Canadian research team was able to demonstrate that more favorable organizational context as assessed by the ACT is positively associated with staff outcomes such as use of best practice (Estabrooks et al., 2015b), lower burnout (Estabrooks et al., 2012), and a better trajectory of resident symptom burden in the last 12 months of life (Estabrooks et al., 2015a). Comparable German research tools were unavailable, therefore we translated the Canadian tools into German, adapted them to the context of German LTC settings, and studied their psychometric properties. In two previously published papers, we report on the translation process (Hoben et al., 2013) and the linguistic validation of the translated tools (Hoben et al., 2014a). Here we report the factor structure (dimensionality), the reliability (based on the results of the factor analyses), and the multiple-group measurement invariance of the translated ACT and CRU Scale.

## Materials and methods

### Framework for psychometric testing: the standards for educational and psychological testing

The Standards for Educational and Psychological Testing (AERA et al., 2014) (hereafter referred to as “*The Standards*”) are recognized as best practice in psychometric testing (Streiner and Norman, 2008). At the time we carried out this study, the 1999 version of *The Standards* (AERA et al., 1999) were the current version. They were the basis for validating the Canadian versions of the ACT and the CRU Scale (Squires et al., 2011a, 2013b; Estabrooks et al., 2011b) and were used to assess the psychometric properties of similar tools for use in LTC (e.g., Gagnon et al., 2011; Zúñiga et al., 2013). The principles and the reliability and validity concepts outlined in *The Standards* guided our methods here.

*The Standards* define validity as a multi-faceted but unitary concept. Different facets of validity can be evaluated from different sources of validity evidence. Validity then is “the degree to which all the accumulated evidence supports the intended interpretation of test scores for the proposed purpose” (AERA et al., 1999, p. 11). According to *The Standards*, the four sources of evidence for validity of a research tool are defined as follows:

1. **Evidence based on test content**. To generate this kind of validity evidence, researchers evaluate if the instrument contents (concepts, topics, wording and format of the introductory texts, items, scales, etc.) represent the intended constructs. Methods typically used for this purpose are theory- and evidence-based tool development, and evaluation of the tools by content experts. We used rigorous methods of instrument translation based on best practice guidelines, including two independent forward and back translations, forward translation review by a panel of content experts, and back translation review by the tool developers (Hoben et al., 2013).
2. **Evidence based on response processes**. Evaluating response processes helps researchers to find out if participants understand the items as intended, if they understand how to use the tools, and if and why they encounter any difficulties when answering the questions. This kind of validity evidence is typically assessed by linguistic validation methods, such as cognitive interviews with target persons (Willis, 2005). Results of cognitive debriefings of the translated tools based on semi-structured cognitive interviews with care providers are published elsewhere (Hoben et al., 2014a).
3. **Evidence based on internal instrument structure**. These analyses evaluate the extent of the association between instrument items/components and the proposed constructs of the tool. Principal component analyses, exploratory factor analyses, or confirmatory factor analyses are the methods traditionally used here. We report methods and results of this analysis for the German-language versions of the ACT and the CRU Scale in this article.
4. **Evidence based on relations to other variables**. The relationship between the instrument variables and other (external) parameters can be evaluated in many different ways. Methods range from simple bivariate correlations to complex latent variable causal models. These analyses evaluate if the instrument constructs are associated with other constructs as expected, based on available theory and evidence. We are currently working on these analyses for the German-language versions of the ACT and the CRU Scale.

### Measures

#### The alberta context tool

The ACT is based on the Promoting Action on Research Implementation in Health Services (PARiHS) framework (Kitson et al., 1998; Rycroft-Malone, 2004) and related literature (Fleuren et al., 2004; Greenhalgh et al., 2004), and it is constructed to assess modifiable characteristics of organizational context (Squires et al., 2013b). According to the PARiHS framework, organizational context is “the environment or setting in which people receive health care services, or in the context of improving care practices, the environment or setting in which the proposed change is to be implemented” (Rycroft-Malone, 2004, p. 299). The ACT is available in versions for adult acute care, pediatric acute care, LTC, and home care, has been translated into French, Dutch, Swedish, Chinese, and German, and is being used in Canada, the United States, Sweden, the Netherlands, the United Kingdom, the Republic of Ireland, Australia, China, and Germany (Squires et al., 2013b). The Canadian ACT LTC (Estabrooks et al., 2011b) contains questionnaires for six different provider groups: (1) care aides, (2) professional (registered or licensed) nurses, (3) allied health providers, (4) practice specialists (e.g., clinical educators, nurse practitioners, clinical nursing specialists, quality improvement specialists), (5) care managers, and 6) physicians. The 56 to 58 items (depending on the form) reflect 10 concepts of organizational context. These 10 concepts are delineated in Table 1, along with a definition and an example item for each concept.

**Table 1:** **Concepts, definitions and example items of the Canadian Alberta Context Tool Long Term Care version (Estabrooks et al., 2011b)**.

**Concept**	**Definition**	**Sample item**
Leadership^a^	The actions of formal leaders in an organization (unit) to influence change and excellence in practice, items generally reflect emotionally intelligent leadership	The leader calmly handles stressful situations
Culture^a^	The way that “we do things” in our organizations and work units; items generally reflect a supportive work culture	My organization effectively balances best practice and productivity
Feedback^a^	The process of using data to assess group/team performance and to achieve outcomes in organizations or units (i.e., evaluation)	Our team routinely monitors our performance with respect to the action plans
Social Capital^a^	The stock of active connections among people. These connections are of three types: bonding, bridging, and linking	People in the group share information with others in the group
Informal Interactions^b^	Informal exchanges that occur between individuals working within an organization (unit) that can promote the transfer of knowledge	[How often do you interact with] people in the following roles or positions? - Someone who champions research and its use in practice
Formal Interactions^b^, ^c^	Formal exchanges that occur between individuals working within an organization (unit) through scheduled activities that can promote the transfer of knowledge	How often do these activities occur? - Team meetings
Structural/Electronic Resources^c^	The structural and electronic elements of an organization (unit) that facilitate the ability to assess and use knowledge	How often do you use/attend the following? - Notice Boards
Organizational Slack (OS)	The cushion of actual or potential resources which allows an organization (unit) to adapt successfully to internal pressures for adjustments or to external pressures for changes	
OS Staff^a^		Enough staff to deliver quality care
OS Space^a^		Use of designated space
OS Time^a^		Time to do something extra for residents

a *Scale 1: (1) strongly disagree, (2) disagree, (3) neither agree or disagree, (4) agree, (5) strongly agree*.

b *Scale 2: (1) never, (2) rarely, (3) occasionally, (4) frequently, (5) almost always*.

c *Scale 3: As scale 2, plus (6) not available*.

Estabrooks et al. (2009b) describe the development and initial validation of the Canadian ACT. Validity of instrument contents was established by the tool developers who were all content experts in the respective fields (Squires et al., 2013b). Response process validity evidence was assessed for all four ACT versions (Squires et al., 2013b). To date, eight studies providing information on the reliability and validity of the ACT have been published (Table 2). Estabrooks et al. (Estabrooks et al., 2009b) found that the pediatric acute care version was acceptably reliable, had a 13-factor structure based on principle component analyses (evidence based on internal instrument structure), and that the ACT concepts were significantly associated with instrumental research utilization (evidence based on relationships with other variables). Furthermore, the instrument developers demonstrated that the ACT scores obtained from individual participants could be validly aggregated at the unit level. This provided evidence for the correlation of individual ACT observations within hospital units. These findings could be confirmed for the ACT LTC version, based on responses from care aides (Estabrooks et al., 2011b), and for the ACT questionnaire for nurses, based on combined results of five studies conducted in LTC, acute adult and pediatric hospitals, and community/home care (Squires et al., 2015). However, the hypothesized 10-factor structure was not confirmed by the confirmatory factor analyses (CFA) (Estabrooks et al., 2011b; Squires et al., 2015). The relative fit indices of the 10-factor model and the two additional models (one including the seven scale-based concepts, and the other including the three count-based concepts) suggested best fit for the model including the seven scale-based concepts. χ^2^ tests of all models were significant, which the authors expected, as the ACT was not developed as a factor model. Using hierarchical linear models, the authors also demonstrated that the ACT pediatric acute care (Estabrooks et al., 2011c) and the ACT LTC (Estabrooks et al., 2011a) were able to discriminate between different care units. Most recent evidence based on multi-level models suggests that the ACT concepts are significant predictors of research utilization (Squires et al., 2013a; Estabrooks et al., 2015b) as well as resident outcomes (Estabrooks et al., 2015a; relation to other variables validity evidence).

**Table 2:** **Overview of studies examining the reliability and validity of the Alberta Context Tool**.

**Study**	**Sample**	**Reliability**	**Internal Structure**	**Relationships**	**Aggregation**
Estabrooks et al. (2009b)	7 pediatric hospitals from 6 Canadian provinces 752 nurses	Cronbach's α < 0.70 for four concepts (Formal Interactions, Resources Type 2 [Traditional], Resources Type 3 [Electronic], OS Space) 0.70–0.91 for all other concepts	Principal component analysis 13-factor solution, accounting for 59,26% of the variance in organizational context	Bivariate correlations between ACT scores and IRU Significant (*p* < 0.01) correlations of all ACT scores with IRU 1-way ANOVAs with ACT scores as dependent variables and IRU as factor Increasing ACT scores are associated with increasing IRU (*p* < 0.01)	ANOVAs with ACT scores as dependent variables and unit as factor; calculated ICC1, ICC2, η^2^, and ω^2^, based on the ANOVA parameters All ANOVAs significant (*p* < 0.001); calculated parameters justified aggregation of individual scores on unit level
Estabrooks et al. (2011c)	32 units in 8 pediatric hospitals 844 nurses	—	—	Hierarchical linear models (random intercept, fixed effects); 40 models (four for each ACT concept) were estimated in a stepwise approach: a) null-model, b) individual predictors (level 1), c) individual predictors (level 1) and specialty predictors (level 2), d) individual predictors (level 1) and specialty and other unit predictors (level 2) Found evidence of relationships between a variety of individual and unit-level variables; specialty and unit level variables explained additional variance in 6 and 7 of the 10 ACT variables, respectively	ANOVAs with ACT scores aggregated on unit level as dependent variable and (a) unit, and (b) specialty of unit as factor (a) All ACT concepts differed between the units (*p* < 0.0001) (b) Except for Informal Interactions, Social Capital, and Resources, ACT concepts differed between specialties (p between 0.018 and < 0.0001) Caterpillar plots (unit-level aggregated ACT scores and 95% confidence intervals) also demonstrated differences in all ACT scores between the units
Estabrooks et al. (2011b)	25 LTC facilities 645 care aides	Cronbach's α < 0.70 for two concepts (Formal Interactions, OS Space) 0.70 to −0.92 for all other concepts	Confirmatory factor analysis, three models: a) all ten factors, b) seven scale-based factors (items rated using an agreement scale ranging from 1 strongly disagree to 5 strongly agree), and c) three count-based factors (items rated using a scale ranging from 1 never to 5 almost always) Model a): χ^2^ = 4674 (df = 1550, *p* < 0.0001), RMSEA = 0.06, SRMR = 0.07, CFI = 0.91 Model b): χ^2^ = 933 (df = 474, p = 0.0001), RMSEA = 0.04, SRMR = 0.04, CFI = 0.98 Modell c): χ^2^ = 828 (df = 176, *p* < 0.0001), RMSEA = 0.09, SRMR = 0.08, CFI = 0.85	Bivariate correlations between the ACT concepts and IRU Except for OS Staffing and OS Space, all ACT concepts were significantly (*p* < 0.01) correlated with IRU (coefficients between 0.111 and 0.199) 1-way ANOVAs with ACT scores as dependent variables and IRU as factor and OS Space, increasing ACT scores are significantly associated with increasing IRU scores (p between 0.03 and < 0.0001)	ANOVAs with ACT scores as dependent variables and a) facility, and b) unit as factor; calculated ICC1, ICC2, η^2^, and ω^2^, based on the ANOVA parameters a) Facility level: Except for Formal and Informal Interactions, all ANOVAs were significant (*p* < 0.0001); calculated parameters justified aggregation of individual scores on facility level b) Unit level: Except for Formal and Informal Interactions, all ANOVAs were significant (*p* between 0.006 and < 0.0001); calculated parameters justified aggregation of individual scores on unit level
Estabrooks et al. (2011a)	89 units in 25 LTC facilities 1258 care aides	—	—	2- and 3-level hierarchical linear models; three null models for each ACT concept: a) unit as level 2 cluster variable, b) facility as level 2 cluster variable, c) unit as level 2 and facility as level 3 cluster variable Significantly (*p* < 0.05) higher percentage of variance in the ACT concepts explained at unit level compared to individual and/or nursing home levels (between +0.72% and +3.3%)	ANOVAs with ACT scores as dependent variables and a) facility, and b) unit as factor; calculated ICC1, ICC2, η^2^, and ω^2^, based on the ANOVA parameters a) All ANOVAs were significant (p between 0.002 and < 0.0001); calculated parameters justified aggregation of individual scores on facility level b) All ANOVAs were significant (p between 0.034 and < 0.0001); calculated parameters justified aggregation of individual scores on unit level
Squires et al. (2013a)	32 units in 8 pediatric hospitals 735 nurses	—	—	General Estimating Equations accounting for correlations of individual ACT scores of RNs working on the same unit; also included various staff level outcomes; independent variables were IRU and CRU Culture (*p* < 0.05) was a significant predictor of IRU Significant predictors of CRU were: Leadership (*p* < 0.001), Culture (*p* < 0.05), Feedback (*p* < 0.001), Formal Interactions (*p* < 0.001), Informal Interactions (*p* < 0.001), and OS Space (*p* < 0.05)	ANOVAs with ACT scores as dependent variables and unit as factor; calculated ICC1, ICC2, η^2^, and ω^2^, based on the ANOVA parameters All ANOVAs significant (*p* < 0.001); calculated parameters justified aggregation of individual scores on unit level
Estabrooks et al. (2015a)	36 LTC facilities 1381 care aides 3647 residents	—	—	Facilities were grouped into a high and a low context group, based on their scores of the ten ACT concepts using *k*-means clustering Trajectories of 6 burdensome symptoms (dyspnea, pain, pressure ulcers, urinary tract infections, challenging behavior, delirium) and one inappropriate practice (use of antipsychotics without diagnosis of psychosis) at the end of life of NH residents were assessed with the Resident Assessment Instrument-Minimum Data Set, version 2.0 Hierarchical mixed model, repeated measures regression, to simultaneously evaluate effects of time, dementia, and context on symptom trajectories High context facilities had significantly lower prevalence of dyspnea, pain and urinary tract infections, and higher prevalence of challenging behavior and delirium but lower use of antipsychotics without diagnosis of psychosis (all *p* < 0.0001)	—
Estabrooks et al. (2015b)	25 LTC facilities 1262 care aides	—	—	Hierarchical linear modeling to identify predictors of HCAs' best practices use Significant ACT predictors of IRU were: Social Capital (*p* < 0.0001), OS Staff (p = 0.005), OS Time (*p* < 0.001, Informal Interactions (p = 0.0005) Significant ACT predictors of CRU were: Feedback (*p* < 0.0001), Structural Resources (p = 0.001), and OS Time (*p* < 0.001)	—
Squires et al. (2015)	2361 nurses from five different study samples: (1) 36 Canadian LTC facilities 325 nurses (2) Canadian acute pediatric hospitals 819 nurses (3) Canadian LTC facilities, acute pediatric hospitals, acute adult hospitals, community/home care 702 nurses (4) Community/home care 348 nurses (5) Australian acute adult hospitals 224 nurses	Cronbach‘s α < 0.70 for one concept (Formal Interactions,0.59) 0.90–0.92 for all other concepts	Confirmatory factor analysis, three models: a) all ten factors, b) seven scale-based factors (items rated using an agreement scale ranging from 1 strongly disagree to 5 strongly agree), and c) three count-based factors (items rated using a scale ranging from 1 never to 5 almost always) Model a): χ^2^ = 13469 (df = 1494, *p* < 0.001), RMSEA = 0.07, SRMR = 0.06, CFI = 0.94 Model b): χ^2^ = 2783 (df = 474, *p* = < 0.001), RMSEA = 0.05, SRMR = 0.04, CFI = 0.98 Modell c): χ^2^ = 7598 (df = 249, *p* < 0.001), RMSEA = 0.13, SRMR = 0.09, CFI = 0.86	Bivariate correlations between the ACT concepts and IRU All ACT concepts were significantly (*p* < 0.001) correlated with IRU (coefficients between 0.093 and 0.262) 1-way ANOVAs with ACT scores as dependent variables and IRU as factor Increasing scores of all ACT concepts are significantly associated with increasing IRU scores (p between 0.004 and < 0.0001) Bivariate correlations between the ACT concepts and CRU All ACT concepts were significantly (*p* < 0.001) correlated with CRU (coefficients between 0.082 and 0.249) 1-way ANOVAs with ACT scores as dependent variables and CRU as factor Except for OS Space, increasing scores of ACT concepts are significantly associated with increasing CRU scores (p between 0.0025 and < 0.0001)	—

*η^2^, Proportion of variance of the variable of interest explained by group membership; ω^2^, Indicator of effect size (relative power of the aggregated variable on sub-group level); ACT, Alberta Context Tool; ANOVA, Analysis of Variance; CFI, Comparative Fit Index; CRU, Conceptual research utilization; ICC1, Interclass Correlation 1 (index indicating clustering of individual ACT scores; possible values range from 0, no clustering to 1, perfect agreement within group); ICC2, Interclass Correlation 2 (index indicating the stability (average reliability) of aggregated data within group; possible values range from 0, no reliability to 1, perfect reliability); IRU, Instrumental Research Utilization; LTC, Long term care; OS, Organizational Slack; RMSEA, Root Mean Square Error of Approximation; RN, Registered Nurse; SRMR, Standard Root Mean Square Residual*.

Information is limited on the psychometric characteristics of translated ACT versions. In addition to the results of the German LTC version (Hoben et al., 2013, 2014a), only results for the Swedish LTC version (Eldh et al., 2013) have been published. The authors report internal consistency reliability (Cronbach's α) values (all >0.70 except for Culture and Informal Interactions) and some validity evidence relating to instrument content and response processes.

#### The conceptual research utilization (CRU) scale

Conceptual Research Utilization is defined as “the cognitive use of research where the research findings may change one's opinion or mind set about a specific practice area but not necessarily one's particular action” (Squires et al., 2011a). Among the different types of research utilization, CRU is of particular importance as it seems to occur more frequently than other types or research utilization (e.g., instrumental = direct application of research knowledge in bed-side care or symbolic = using research to justify own activities or to convince others to change their actions) and therefore “is believed to be more reflective of the process of research utilization at the individual practitioner level” (Squires et al., 2011a). Policy makers and knowledge users frequently do not use research to *act* upon a situation, but rather to *inform* their decision making (Squires et al., 2011a).

To assess CRU, researchers need a robust research tool that can be validly used with various provider groups (particularly care aides) in LTC settings. Squires et al. (2011a) developed the CRU Scale for this purpose. The five-item tool is available in two versions: one for care aides and one for regulated care providers. Participants are asked how often research had five different specific effects on their last typical day of work e.g., “Help to change your mind about how to care for residents.” Participants rate each of the five items on a scale ranging from “1 = Never” (care aides) or “10% or less of the time” (regulated providers) to “5 = Almost Always” (care aides) or “Almost 100% of the time” (regulated providers).

The psychometric properties of the CRU Scale were comprehensively assessed based on *The Standards* (Squires et al., 2011a). Validity of the instrument content was evaluated in a formal process with a sample of nine international research utilization experts. Response processes were studied with ten care aides from two LTC facilities. In a third sample of 707 care aides from 30 LTC facilities, the authors assessed the reliability of the CRU Scale (Cronbach's α = 0.89, Guttman Split Half = 0.86, Spearman-Brown = 0.89). The internal structure of the scale was assessed using CFA. Removal of one sub-optimal item and correlation of residual variances led to a well-fitting four-item one-factor model: χ^2^ = 2.43, df = 1, *p* = 0.119, RMSEA = 0.045, SRMR = 0.007, CFI = 0.999. For the five-item CRU version we used in this study, the tool developers had revised this sub-optimal item. Finally, the relationships between CRU and other types of research utilization were assessed using bivariate correlations (significant correlations for the CRU Scale score and all five CRU items) and multivariate linear regression (CRU Scale score was a significant predictor of overall research utilization).

The authors also assessed the precision of the CRU Scale using item response theory models (Squires et al., 2014). While the scale demonstrated acceptable precision at low and average trait levels (i.e., an individual's levels of CRU), the included items are less optimal in reflecting higher trait levels.

### Sample

#### Facility sample

Our study population included all 251 LTC facilities in one German region (*Metropolregion Rhein-Neckar*) recognized as a residential elder care facility by the German social law (XIth German social security statute book–*SGB XI*). These facilities were stratified by three criteria (Table 3).

1. Federal state: Baden-Württemberg, Hessia, Rhineland-Palatinate.
2. Size: small (≤ 60 beds), medium (61–120 beds), large (>120 beds).
3. Provider type: voluntary not-for-profit, public not-for-profit, private for-profit.

**Table 3:** **Residential long term care facilities in the German region *Metropolregion Rhein-Neckar***.

	**Baden-Württemberg**				**Hessia**				**Rhineland-Palatinate**				**Total**			
	**Small**	**Medium**	**Large**	**Total**	**Small**	**Medium**	**Large**	**Total**	**Small**	**Medium**	**Large**	**Total**	**Small**	**Medium**	**Large**	**Total**
Voluntary Not-For-Profit	18	38	15	71	2	6	3	11	5	26	10	41	25	70	28	123
Public Not-For-Profit	0	2	1	3	0	0	1	1	1	2	2	5	1	4	4	9
Private For-Profit	32	21	6	59	13	4	3	20	10	15	15	40	55	40	24	119
Total	50	61	22	133	15	10	7	32	16	43	27	86	81	114	56	251

*Small: ≤ 60 beds, Medium: 61–120 beds, Large: >120 beds*.

An independent person not involved in other parts of this study drew a stratified random sample of 38 facilities from this pool of facilities. In each of the 27 categories (3 provider types × 3 size categories × 3 federal states) one facility was randomly selected. In each of the three federal states two additional facilities were drawn for the categories “medium, voluntary not-for-profit” and “large, voluntary not-for-profit,” because facilities of these categories are also over-represented in the overall population of German national LTC facilities (Statistisches Bundesamt, 2013). In the case of empty categories (e.g., Baden-Württemberg, small, public not-for-profit), we included facilities of a similar category (e.g., Baden-Württemberg, small, voluntary not-for-profit). If a selected facility was approached but declined to participate in our study, another facility of the same category was drawn. If, in any one federal state, all facilities in a category and in similar categories declined to participate, facilities in the same category from another federal state were contacted.

#### Provider sample

Eligible participants for this study were all care providers (care aides, nurses, allied providers), nursing students (who complete the care aide survey), clinical specialists, and managers who met the inclusion criteria:

1. Employed in one of the included LTC facilities.
2. Have been working on their unit (care aides, nurses, students, care managers) or in the facility (allied providers, specialists, directors of care, facility managers) for ≥3 months (this criterion was applied in previous usage of the ACT as providers need to have sufficient time on the unit to report on the unit context).
3. Work at least 25% of the hours of a full-time job.
4. Able to read and write in German.

Voluntary workers or casual staff were not eligible.

### Data collection

At one-day data collection appointments, a researcher distributed questionnaires to all eligible persons present during the morning and evening shifts at the facility. The participants filled out the questionnaires during their work time and returned the completed questionnaires to the researcher. The researcher was available for questions and concerns at all times while participants completed the questionnaires.

### Data analysis

#### Missing data, distribution and descriptive statistics

We used SPSS version 20 (IBM, 2011) to analyze missing data, normal distribution of variables, and descriptive statistics. Except for an answer to item five of the CRU Scale, which was missing in 25 questionnaires (3%), answers to all ACT and CRU Scale items were missing in less than 3% of the questionnaires. According to the *Missing Completely at Random (MCAR) Test* (Little and Rubin, 2002) (χ^2^ = 12,426.806, df = 12,434, *p* = 0.517) missing items were distributed completely at random. This indicates that no systematic problems caused the missing answers (Graham, 2012), therefore we removed three questionnaires with >25% of data missing (one each of the care aide, nurse, and allied questionnaires). Missing data were not imputed, but deleted listwise in the analyses.

Our analyses indicated that none of the variables were distributed normally. Skewedness and kurtosis values were substantially different from zero, and their values were clearly more than twice the values of their corresponding standard errors (Miles and Shevlin, 2001). The Kolmogorov-Smirnov Test (Kolmogorov, 1933; Smirnov, 1948) and the Shapiro-Wilk Test (Shapiro and Wilk, 1965) supported those findings; the distribution of all ACT and CRU Scale variables was significantly different from normal (*p* < 0.0001). However, descriptive analyses indicated no extreme outliers (> 3 × interquartile range).

#### Factor structure and measurement invariance

We assessed the internal structures of the ACT and the CRU Scale, using CFA. We tested whether the factor structure of the translated tool was the same as the factor structure proposed for the Canadian versions. Further, we assessed the measurement invariance of the translated tools across the different provider groups, using multiple-group CFA models. Measurement invariance analyses evaluate if the constructs of a tool are measured equally well in different groups or if their measurement differs substantially (Byrne et al., 1989; Brown, 2006; Dimitrov, 2010; Sass, 2011; Wang and Wang, 2012). We could not run factor models in the specialist sub-sample, as this sample only included six participants. However, we included the responses of the six specialists in the models of the entire sample. We used Mplus Version 7.11 (Muthén and Muthén, 2013) for these analyses. Due to the non-normality of our data we used a robust mean- and variance-adjusted maximum likelihood estimator (MLMV) (Asparouhov and Muthén, 2010). Unless reported otherwise, we fixed the first item loading of each factor to 1.0 in all models to identify the models (default setting in Mplus) (Muthén and Muthén, 2012).

**Figure 1:**
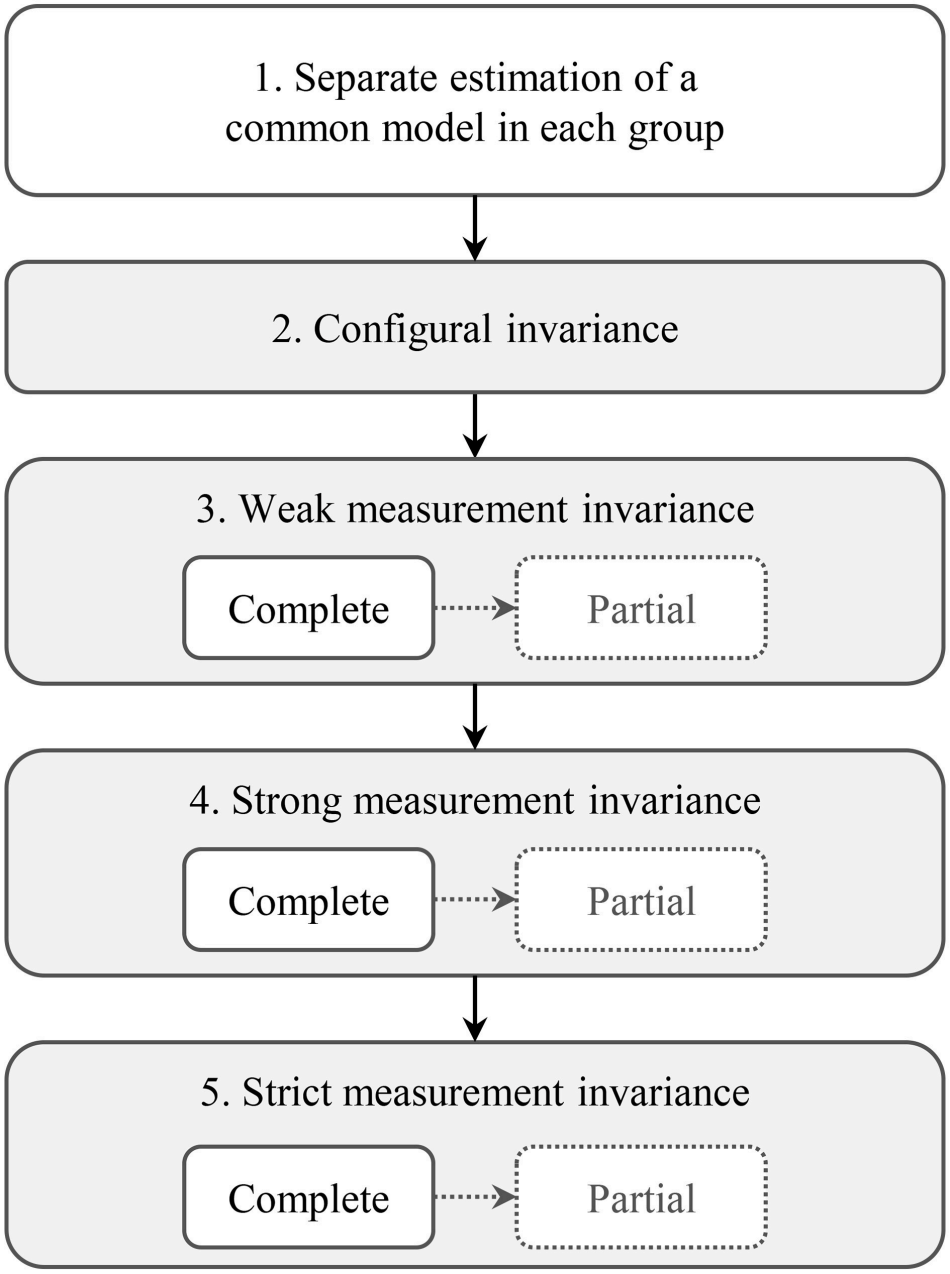
**Steps of the measurement invariance analysis**.

The items related to each ACT concept are explicitly developed to be non-redundant, although they capture similar aspects of that context feature (Estabrooks et al., 2011b). With these similarities of items, the factor structure is still the most appropriate of the available model structures, even if it is not a 100% perfectly specified model (Estabrooks et al., 2011b). Model fit evaluation followed well-established recommendations (Brown, 2006; Raykov and Marcoulides, 2006; Kline, 2011a; Wang and Wang, 2012; West et al., 2012). We report the χ^2^ test, as this is the only statistical test available to determine the consistency between the model-implied covariance matrix (from the CFA model) and the sample covariance matrix (from our data); a non-significant χ^2^ value (*p*>0.05) implies no detectable ill fit (Hayduk, 1996; Hayduk and Glaser, 2000; Hayduk et al., 2007). We also report common “close-fit” indices: (a) Root Mean Square Error of Approximation (RMSEA), (b) Comparative Fit Index (CFI), (c) Tucker Lewis Index (TLI), and d) Standardized Root Mean Square Residual (SRMR). These are independent of the sample size. Our interpretation of these indices followed the recommendations of Hu and Bentler (Hu and Bentler, 1999) who suggested cut-off values for each index based on simulation studies: RMSEA < 0.06, CFI and TLI > 0.95, and SRMR < 0.08. In addition to this global evaluation of model fit, we also investigated models for local areas of strain. We evaluated the estimated model parameters (e.g., statistical significance, expected size and direction of loadings, intercepts, residual variances), and the modification indices (e.g., evidence of misspecifications and suggestions for model modifications; Brown, 2006; Raykov and Marcoulides, 2006; Kline, 2011a; Wang and Wang, 2012; West et al., 2012). In assessing measurement invariance, we followed the steps outlined in Figure 1 (Byrne et al., 1989; Brown, 2006; Dimitrov, 2010; Sass, 2011; Wang and Wang, 2012).

##### Step 1: separate estimation of a common model in each group

**Figure 2:**
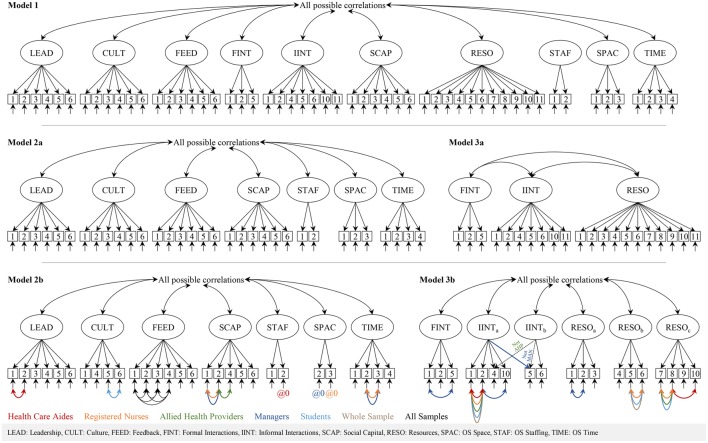
**Alberta Context Tool factor models**.

Our first step was to find a model that properly fit each of the different participant groups. We therefore ran all models separately on each of the sub-samples (care aide, nurse, allied providers, managers, students) and on the entire sample. In our analyses we included only items that are available in all questionnaire versions, as comparable models with the same structure are required to run multiple-group CFA. Therefore, we removed the eight group-specific items (Organizational Slack [OS] Staffing, item 3; Formal Interactions, items 3 and 4; Informal Interactions, items 3, 7-9, and 12). The first model (Figure 2, model 1) included all ten ACT concepts. We also decided a priori to run two separate models for the scale-based and the count-based ACT concepts (Figure 2, models 2a, 3a) as suggested by Estabrooks et al. (2011b). We allowed residual variances of items of the same concept to correlate in specific sub-samples if (a) suggested by the modification indices and (b) item contents and experiences during the instrument translation and pretesting or during the data collection of this study justified this. For rationales in correlating residual variances see Supplementary Table 1.

**Figure 3:**
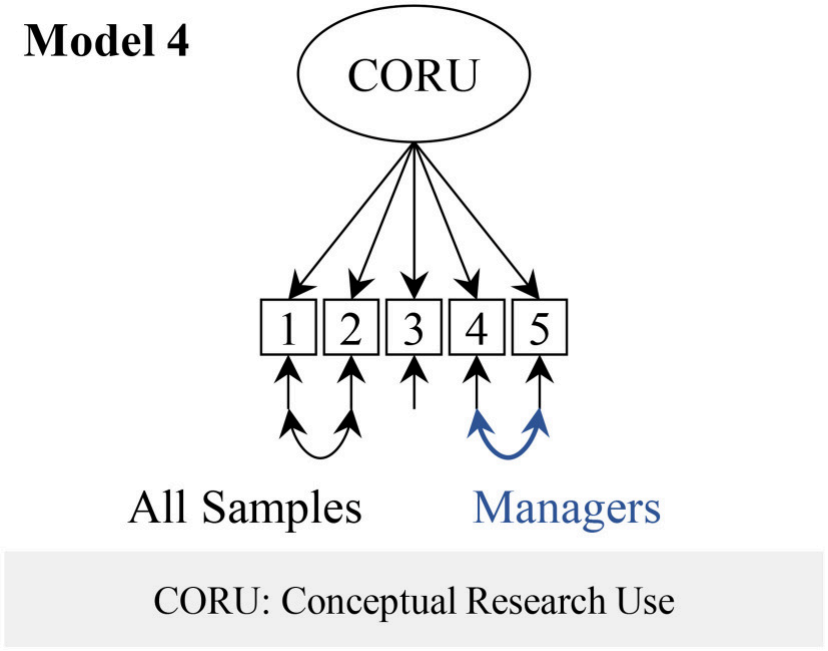
**Conceptual Research Utilization Scale factor models**.

We ran a one-factor model of the CRU Scale (Figure 3, model 4) as suggested in the validation study of the original Canadian tool (Squires et al., 2011a).

##### Step 2: configural invariance

We estimated each of the three models (2b, 3b, and 4) simultaneously in all five provider group sub-samples. All model parameters were freely estimated in each group.

##### Step 3: weak measurement invariance

In this step we assessed if the associations (loadings) between the latent factors and the related indicators (items) were the same across the provider groups. We restrained the loadings to be equal across the five provider groups, with all other model specifications remaining the same as in the previous model (configural invariance). If the item loadings are the same across the groups, a change in the latent construct (e.g., the ACT factor TIME) by one unit changes the value of the related items (e.g., the ACT Time item “How often do you have time to do something extra for residents?”) equally strongly and in the same direction in all five groups. Weak measurement invariance allows group comparisons of associations between latent factors and external variables, and of factor variances and covariances (Byrne et al., 1989; Brown, 2006; Dimitrov, 2010; Sass, 2011; Wang and Wang, 2012). However, structural coefficients of the model and factor means cannot be compared (Byrne et al., 1989; Brown, 2006; Dimitrov, 2010; Sass, 2011; Wang and Wang, 2012). Equal loadings indicate a common scale across the groups, but we do not know if the starting point of this scale is the same in all groups.

Weak measurement invariance was noted if the fit of the restrained model (equal loadings) was not significantly worse than the fit of the previous (configural invariance) model. We assessed this using the χ^2^ difference test (p value expected to be >0.05). The MLMV estimator in Mplus uses the DIFFTEST function for this, which produces a corrected χ^2^ difference test (Muthén and Muthén, 2012). If weak measurement invariance was established for a latent factor, we continued with step 4. Otherwise we freed individual loadings sequentially, starting with the loading with the highest modification index value. If less than 20% of the loadings in the model needed to be freed to gain a non-significant χ^2^ difference test, partial weak measurement invariance was accepted (Byrne et al., 1989; Vandenberg and Lance, 2000; Dimitrov, 2010) and we continued with step 4. Otherwise we stopped the procedure here.

##### Step 4: strong measurement invariance

Based on the previous model (equal loadings except for the freed ones) we now restrained the item intercepts to be equal across the groups. An item intercept can be interpreted as the predicted item value if the score of the latent construct is zero (Brown, 2006). If the item intercepts differ across the groups, groups with the same value of the latent construct (e.g., the ACT factor TIME) on average give different answers to the related items (e.g., the ACT Time items). This is called item bias or differential item functioning (Byrne et al., 1989; Brown, 2006; Dimitrov, 2010; Sass, 2011; Wang and Wang, 2012). If a tool is strongly measurement invariant, factor means and structural coefficients of the model can be compared across groups. Again, this model was compared to the previous model (weak measurement invariance). If strong measurement invariance was established we continued with step 5, otherwise we freed intercepts sequentially according to the previously described procedure. If partial strong measurement invariance could not be established we stopped our procedure here.

##### Step 5: strict measurement invariance

Based on the (partial) strong measurement invariance model we now restrained the residual variances to be equal across the groups. We then freed residual variances again until strict or partial strict measurement invariance was established or had to be rejected. If residual variances differ across the participant groups, this means that the constructs are measured with different precision (contain different amounts of measurement error) in the different groups. Group differences, therefore, are not only caused by differing “true scores” (i.e., the latent factor scores), but also by differing errors. To compare values of observed variables or scores derived from these values across groups, strict measurement invariance is required (Dimitrov, 2010; Sass, 2011).

#### Reliability

Cronbach's α (internal consistency reliability) is the most commonly reported index to assess reliability, although numerous publications discuss various problems with this index (see Green and Yang, 2009; Sijtsma, 2009; Yang and Green, 2011 for an overview). In particular, two assumptions required for valid results of Cronbach's α are rarely met in practice: (a) essential tau equivalence (all items of a scale have the same loadings on the corresponding factor), and (b) uncorrelated residual variances. If those requirements are not met, Cronbach's α values will be biased. Therefore, the internal consistency reliability of a scale should be assessed using a more robust method based on latent variable models (Green and Hershberger, 2000; Raykov and Shrout, 2002; Green and Yang, 2009; Yang and Green, 2011). As Cronbach's α is a popular index and for comparability reasons, we also report Cronbach's α. however, we are relying on a more robust reliability index (ω in the following). According to this approach, reliability is the ratio of the true score variance (σ_true_) and the total variance (σ_tot._) of a scale. Total variance of a scale (σ_tot_) is the sum of σ_true_ and the residual variance σ_res._ of the scale. From this it follows that ω = σ_true_/(σ_true_ + σ_res._). As each ACT concept represents a distinct construct, we calculated a separate reliability score for each. Therefore, σ_true_ is the squared sum of the loadings of the respective factor and σ_res_. is the sum of the residual variances of the corresponding items. For the reliability calculations we used the individual factor models (2b, 3b, and 4). As we needed to estimate all loadings in these models, we freed the first loading of each factor (previously fixed to 1), and instead fixed the factor variances to zero in order to obtain an identified model.

### Ethics approval

The study was approved in writing by the ethics board of the Medical Faculty, Martin-Luther University Halle-Wittenberg, Halle (Saale), Germany (reference number: 2011-39). All participants completed written informed consent before participation.

## Results

### Sample

We contacted 133 facilities and 41 agreed to participate, but three canceled their participation before start of data collection due a shortage of staff. Our participation rate was therefore 28.6% of facilities contacted. Reasons given by facilities for not participating were: (a) other projects (e.g., implementation of new care documentation software or participation in other research projects; *n* = 37), (b) staff shortage (*n* = 27), (c) no senior person available to make this decision (e.g., due to a change of leadership; *n* = 11), (d) staff surveys unwelcome (*n* = 6), (e) no reasons stated (*n* = 5), (f) refusal by works council or staff (*n* = 3), team problems (*n* = 2), and (h) bad experiences with previous research projects (*n* = 1).

Overall, we retrieved 824 questionnaires from six different provider groups (Table 4). This is a response rate of 37.7% for all potentially eligible persons employed in the 38 participating facilities (response rate 1). However, we could not approach all these potentially eligible persons during our data collection appointments. Based on the number of eligible persons available during our data collection appointments, our response rate was 81.5% (response rate 2).

**Table 4:** **Response rates overall and by provider groups**.

	**Questionnaires**		**Response Rate 1 (%)**				**Response Rate 2 (%)**			
	***N***	**%**	**Overall**	**Min**.	**Max**.	***SD***	**Overall**	**Min**.	**Max**.	***SD***
Care aides	274	33.3	33.8	1.0	10.0	2.5	76.1	28.6	77.6	19.0
Nurse	197	23.9	31.6	9.1	8.0	14.0	8.7	5.0	82.3	15.5
Allied	153	18.6	5.2	14.3	10.0	23.7	81.4	25.0	83.3	22.5
Specialist	6	0.7	6.0	0.0	10.0	51.6	10.0	10.0	10.0	0.0
Managers	129	15.7	73.7	33.3	10.0	2.5	92.8	5.0	93.9	12.3
Students	65	7.9	25.1	0.0	85.7	21.2	87.8	0.0	91.7	21.0
Overall	824	100	37.7	23.3	79.3	11.2	81.5	56.7	82.9	11.7

*Response Rate 1, number of questionnaires collected/number of potentially eligible persons employed in the facility; Response Rate 2, number of questionnaires collected/number of eligible persons available at data collection*.

Three questionnaires were excluded due to extensive missingness as described in the methods section, leaving us with 821 questionnaires. Table 5 displays the socio-demographic characteristics of the participants. The characteristics of sex, age, native language, percentage of a full-time job, and general education differed significantly (p < 0.0001) between the provider groups. Job training is specific to each group, thus not analyzed across groups. All care aides, nurses, care managers, and nursing students were assigned to a certain unit in their facility, and all specialists and other managers were working across the entire facility, except in the allied group. Of the 152 allied providers, 101 were working across various units in their facility and 51 were permanently assigned to one unit.

**Table 5:** **Socio-demographic characteristics of the included participants**.

	**Care aides(*N* = 273)**		**Nurses(*N* = 196)**		**Allied(*N* = 152)**		**Specialists(*N* = 6)**		**Managers(*N* = 129)**		**Students(*N* = 65)**		**All(*N* = 821)**	
	***N***	**%**	***N***	**%**	***N***	**%**	***N***	**%**	***N***	**%**	***N***	**%**	***N***	**%**
SEX^***^														
Female	249	91.2	161	82.1	131	86.2	4	66.7	95	73.6	54	83.1	694	84.5
Male	24	8.8	35	17.9	20	13.2	2	33.3	34	26.4	11	16.9	126	15.4
*Missing*	—	—	—	—	*1*	*0.7*	—	—	—	—	—	—	*1*	*0.1*
AGE^***^														
≤ 19 Years	15	5.5	—	—	3	2.0	—	—	—	—	12	18.5	30	3.7
20–29 Years	33	12.1	54	27.6	18	11.8	2	33.3	13	1.1	33	5.8	153	18.6
30–39 Years	52	19.1	47	24.0	17	11.2	2	33.3	23	17.8	9	13.9	150	18.3
40–49 Years	81	29.7	39	19.9	46	3.3	2	33.3	47	36.4	7	1.8	222	27.0
50–59 Years	75	27.5	39	19.9	55	36.2	—	—	44	34.1	2	3.1	215	26.2
>59 Years	12	4.4	10	5.1	11	7.2	—	—	—	—	—	—	33	4.0
*Missing*	*5*	*1.8*	*7*	*3.6*	*2*	*1.3*	—	—	*2*	*1.6*	*2*	*3.1*	*18*	*2.2*
NATIVE LANGUAGE^***^														
German	179	65.6	150	76.5	140	92.1	5	83.3	113	87.6	50	76.9	637	77.6
Not German	90	33.0	45	23.0	12	7.9	1	16.7	15	11.6	15	23.1	178	21.7
*Missing*	*4*	*1.5*	*1*	*0.5*	—	—	—	—	*1*	*0.8*	—	—	*6*	*0.7*
**JOB TRAINING**														
None	188	68.9	—	—	50	32.9	—	—	—	—	—	—	238	29.0
Geriatric Care Aide	45	16.5	—	—	—	—	—	—	—	—	—	—	45	5.5
Acute Care Aide	28	1.3	—	—	—	—	—	—	—	—	—	—	28	3.4
Nurse, not recognized^a^	10	3.7	—	—	—	—	—	—	—	—	—	—	10	1.2
Other^b^	2	0.7	—	—	16	1.5							18	2.2
Geriatric Nurse	—	—	144	73.5	—	—	2	33.3	21	16.3	—	—	167	2.3
Adult Acute Care Nurse	—	—	47	24.0	—	—	—	—	6	4.7	—	—	53	6.5
General Nurse	—	—	3	1.5	—	—	—	—	—	—	—	—	3	0.4
Remedial Care Attendant	—	—	2	1.0	—	—	—	—	—	—	—	—	2	0.2
Recreational Therapist	—	—	—	—	10	6.6	—	—	—	—	—	—	10	1.2
Physiotherapist	—	—	—	—	2	1.3	—	—	—	—	—	—	2	0.2
Social Work (academic)	—	—	—	—	9	5.9			3	2.3	—	—	12	1.5
Social Work (vocational)	—	—	—	—	1	0.7	—	—	—	—	—	—	1	0.1
Dementia Care Assistant^c^	—	—	—	—	64	42.1	—	—	—	—	—	—	64	7.8
Academic Degree in Nursing	—	—	—	—	—	—	1	16.7	6	4.7	—	—	7	0.9
Continuing Education in QI	—	—	—	—	—	—	3	5.0	—	—	—	—	3	0.4
Continuing Education in Management	—	—	—	—	—	—	—	—	89	69.0	—	—	89	1.8
Academic Degree in BA	—	—	—	—	—	—	—	—	3	2.3	—	—	3	0.4
Academic Degree in Education	—	—	—	—	—	—	—	—	1	0.8	—	—	1	0.1
Nursing Student 1st Year	—	—	—	—	—	—	—	—	—	—	23	35.4	23	2.8
Nursing Student 2nd Year	—	—	—	—	—	—	—	—	—	—	21	32.3	21	2.6
Nursing Student 3rd Year	—	—	—	—	—	—	—	—	—	—	20	3.8	20	2.4
*Missing*	—	—	—	—	—	—	—	—	—	—	*1*	*1.5*	*1*	*0.1*
% Of A FULL-TIME JOB^***^														
25–49%	12	4.4	5	2.6	14	9.2	2	33.3	—	—	—	—	33	4.4
50–75%	52	19.1	25	12.8	57	37.5	1	16.7	2	1.6	—	—	137	18.1
>75%	202	74.0	164	83.7	77	5.7	2	33.3	125	97.0	—	—	570	75.4
*Missing*	*7*	*2.6*	*2*	*1.0*	*4*	*2.6*	*1*	*16.7*	*2*	*1.6*	—	—	*16*	*2.1*
GENERAL EDUCATION^***^														
No Certificate/Degree	5	1.8	—	—	2	1.3	—	—	—	—	1	1.5	8	1.0
Secondary School (Grade 9)	49	18.0	—	—	9	5.9	—	—	—	—	13	2.0	71	8.7
Secondary School (Grade 10)	29	1.6	—	—	13	8.6	—	—	—	—	27	41.5	69	8.4
Vocational Training	158	57.9	160	81.6	95	62.5	5	83.3	79	61.2	18	27.7	515	62.7
High School (Grade 12 or 13)	15	5.5	26	13.3	19	12.5	—	—	30	23.3	4	6.2	94	11.5
Academic Degree	9	3.3	9	4.6	13	8.6	1	16.7	19	14.7	2	3.1	53	6.5
*Missing*	*8*	*2.9*	*1*	*0.5*	*1*	*0.7*	—	—	*1*	*0.8*	*0*	*0.0*	*11*	*1.3*
	**M *(SD)***		**M *(SD)***		**M *(SD)***		**M *(SD)***		**M *(SD)***		**M *(SD)***		**M *(SD)***	
Job Experience (Years)^***^	1.82 (9.73)		14.99 (9.62)		5.13 (6.31)		3.33 (1.83)		8.41 (6.66)		3.73 (4.79)		9.78 (9.17)	
Experience in Facility (Years)^***^	7.07 (7.60)		6.81 (5.95)		5.58 (6.43)		4.12 (3.94)		9.49 (7.97)		2.41 (2.65		6.71 (6.98)	

*** *Significant difference (p < 0.001) between the six provider groups (Kruskal-Wallis Test)*.

a *Training as registered nurse in another country, not recognized in Germany*.

b *Examples are: Elder Care Therapist, Practice Nurse, Family Care Aide, Home Help, House Keeper, etc*.

c *Continuing education specific for Germany: people are trained to care for residents with dementia with a specific focus on recreational activities in the daily routine; regulated by the German social law (^§^87b SGB XI); QI, Quality Improvement; BA, Business Administration; M, Mean; SD, Standard Deviation*.

The characteristics of the participating facilities are illustrated in Table 6. The absolute numbers of care aides, nurses, care managers, facility level managers, and nursing students differed substantially between small, medium, and large facilities. However, these differences became smaller when taking into account the amount of work time (i.e., care minutes for each provider group per resident day). Only the nurses and the facility level managers differed significantly in staffing minutes per resident day between facilities of different size. Between facilities of different federal states only nursing care minutes per resident day differed significantly (*p* < 0.05), and facilities of different provider types did not differ in any of the provider group characteristics.

**Table 6:** **Characteristics of the participating facilities**.

	**Small (*****N*** = 8**)**		**Medium (*****N*** = 21**)**		**Large (*****N*** = 9**)**		**All (*****N*** = 38**)**	
	***N***	**%**	***N***	**%**	***N***	**%**	***N***	**%**
**FEDERAL STATE**								
Baden-Württemberg	4	5.0	9	42.9	3	33.3	16	42.1
Hessia	—	—	3	14.3	3	33.3	6	15.8
Rhineland-Palatinate	4	5.0	9	42.9	3	33.3	16	42.1
**PROVIDER TYPE**								
Voluntary/Private, Not-For-Profit	3	37.5	17	81.0	5	55.6	25	65.8
Public, Not-For-Profit	3	37.5	1	4.8	2	22.2	6	15.8
Private, For-Profit	2	25.0	3	14.3	2	22.2	7	18.4
	**Median (Range)**		**Median (Range)**		**Median (Range)**		**Median (Range)**	
Units per Facility	1 (1–2)		3 (1–4)		4 (3–7)		3 (1–7)	
Beds per Facility	51 (24–87)		94 (61–120)		145 (120–181)		97 (24–181)	
Beds per Unit	33 (23–60)		25 (20–80)		36 (26–48)		35 (20–80)	
	**Mean *(SD)***		**Mean *(SD)***		**Mean *(SD)***		**Mean *(SD)***	
**STAFFING (NUMBER OF PERSONS)**								
Care Aides^***^	1.38 (4.98)		2.71 (5.42)		32.56 (9.33)		21.34 (9.81)	
Nurses^***^	1.50 (3.07)		15.24 (3.97)		24.44 (6.33)		16.42 (6.56)	
Allied Providers	5.00 (1.85)		8.24 (5.16)		1.22 (3.27)		8.03 (4.53)	
Specialists	0.13 (0.35)		0.38 (0.50)		0.11 (0.33)		0.26 (0.45)	
Care Managers^***^	1.00 (0.93)		2.52 (0.98)		4.11 (1.17)		2.58 (1.45)	
Directors of Care & Facility Administrators^*^	1.88 (0.64)		1.90 (0.30)		2.44 (0.53)		2.03 (0.49)	
Nursing Students^**^	3.38 (3.20)		6.67 (3.77)		1.22 (2.28)		6.82 (4.02)	
**STAFFING (MINUTES PER RESIDENT DAY)**								
Care Aides	51.00 (12.00)		54.00 (8.40)		56.40 (12.60)		54.00 (1.20)	
Nurses^*^	57.60 (12.00)		46.20 (12.00)		44.40 (9.60)		48.60 (12.00)	
Allied Providers	22.20 (14.40)		18.60 (12.00)		16.20 (4.80)		18.60 (11.40)	
Specialists	0.00 (0.60)		0.60 (1.20)		0.00 (0.60)		0.60 (1.20)	
Care Managers	6.00 (5.40)		9.60 (3.60)		1.20 (1.80)		9.00 (4.20)	
Directors of Care & Facility Administrators^***^	13.80 (6.60)		7.20 (1.80)		6.00 (0.60)		8.40 (4.20)	
Nursing Students	4.80 (5.40)		4.20 (2.40)		4.20 (1.80)		4.80 (3.00)	

* *p < 0.05*,

** *p < 0.01*,

*** *p < 0.001; differences between small, medium and big facilities (one-way analyses of variance); Small: ≤ 60 beds, Medium: 60–120 beds, Large: >120 beds; SD, Standard Deviation*.

### Factor structure and reliability

#### Factor structure of the alberta context tool

Table 7 shows the model fit indices of all factor models. Fit of the ACT model 1 (ten factors) was poor and could not be estimated in the student sub-sample. While fit of the ACT model 2a (seven scale based concepts) improved substantially compared to model 1, it was still not acceptable. Fit of ACT model 3a (three count-based concepts) decreased compared to model 1.

**Table 7:** **Model fit indices of the ACT and CRU Scale factor models**.

**Model**	**Provider**	**N**	**χ^2^**	**df**	**p**	**RMSEA (90%CI)**	**CFI**	**TLI**	**SRMR**
ACT 1 (all ten concepts)	Care aides	242	180.981	1333^*a*^	0.0000	0.038 (0.033-0.043)	0.762	0.745	0.100
	Nurses	172	1707.796	1332	0.0000	0.041 (0.035-0.046)	0.714	0.693	0.098
	Allied	136	1678.855	1332	0.0000	0.044 (0.037-0.050)	0.690	0.667	0.110
	Managers	107	1588.735	1333^b^	0.0000	0.042 (0.033-0.050)	0.644	0.618	0.110
	Students	55	Model could not be estimated						
	All	712	3249.087	1332	0.0000	0.045 (0.043-0.047)	0.777	0.671	0.098
ACT 2a (seven scale-based concepts)	Care aides	249	64.619	475^a^	0.0000	0.037 (0.030-0.045)	0.910	0.900	0.068
	Nurses	178	594.444	474	0.0001	0.038 (0.027-0.047)	0.896	0.884	0.073
	Allied	140	616.171	474	0.0000	0.046 (0.035-0.056)	0.851	0.834	0.086
	Managers	115	558.560	475^b^	0.0048	0.039 (0.023-0.052)	0.868	0.853	0.082
	Students	57	564.000	474	0.0027	0.058 (0.036-0.075)	0.767	0.740	0.094
	All	744	1045.062	474	0.0000	0.040 (0.037-0.044)	0.921	0.911	0.055
ACT 3a (three count-based concepts)	Care aides	263	581.375	186	0.0000	0.090 (0.082-0.098)	0.552	0.495	0.148
	Nurses	183	54.515	186	0.0000	0.102 (0.092-0.112)	0.481	0.414	0.151
	Allied	147	Model could not be estimated						
	Managers	116	428.739	186	0.0000	0.106 (0.093-0.119)	0.509	0.445	0.151
	Students	62	30.198	186	0.0000	0.100 (0.078-0.120)	0.514	0.451	0.159
	All	771	2108.639	186	0.0000	0.116 (0.111-0.120)	0.590	0.537	0.155
ACT 2b (seven scale based concepts, modified model)	Care aides	251	419.795	350	0.0061	0.028 (0.016-0.038)	0.962	0.956	0.053
	Nurses	179	405.821	349	0.0193	0.030 (0.013-0.042)	0.952	0.944	0.059
	Allied	141	401.548	349	0.0273	0.033 (0.012-0.046)	0.945	0.936	0.070
	Managers	118	388.865	349	0.0694	0.031 (0.000-0.047)	0.936	0.926	0.072
	Students	58	388.725	349	0.0701	0.044 (0.000-0.067)	0.894	0.877	0.083
	All	752	602.051	350	0.0000	0.031 (0.027-0.035)	0.964	0.959	0.041
ACT 3b (three count-based concepts, modified model)	Care aides	263	162.869	134	0.0454	0.029 (0.004-0.043)	0.965	0.955	0.055
	Nurses	187	149.882	133	0.1504	0.026 (0.000-0.045)	0.977	0.970	0.045
	Allied	147	178.228	135	0.0075	0.047 (0.025-0.064)	0.930	0.912	0.075
	Managers	117	172.621	132	0.0101	0.051 (0.026-0.071)	0.918	0.893	0.071
	Students	63	145.181	133	0.2219	0.038 (0.000-0.074)	0.948	0.934	0.080
	All	777	313.372	134	0.0000	0.042 (0.036-0.047)	0.961	0.950	0.040
CRU Scale 4	Care aides	268	1.519	4	0.8232	0.000 (0.000-0.055)	0.000	1,006	0.003
	Nurses	183	4.637	4	0.3266	0.030 (0.000-0.199)	0.999	0,997	0.012
	Allied	147	4.682	4	0.3216	0.034 (0.000-0.133)	0.998	0,995	0.010
	Managers	123	3.802	3	0.2837	0.047 (0.000-0.166)	0.998	0,995	0.009
	Students	63	0.297	4	0.9900	0.000 (0.000-0.000)	0.000	1,093	0.005
	All	790	6.207	4	0.1842	0.026 (0.000-0.065)	0.999	0,998	0.005

a *In this model one negative, non-significant residual variance (ST2) was fixed to zero*.

b *In this model one negative, non-significant residual variance (SP2) was fixed to zero; df, degrees of freedom; RMSEA, Root Mean Square Error of Approximation; 90%CI, 90% confidence interval; CFI, Comparative Fit Index; TLI, Tucker Lewis Index; SRMR, Standardized Root Mean Square Error*.

Fit improved substantially with ACT model 2a but inspection still revealed four items substantially contributing to poor model fit: Culture items 2 (member of a supportive work group) and 3 (organization effectively balances best practice and productivity), Social Capital item 3 (other teams share information with my team) and Space item 1 (private space available on this unit or floor). In most or all groups, these items also had substantially smaller loadings than the other items of the same concept (Tables 8–**10**), or high modification indices (indicating, for example, cross-loadings on other than the expected factors. We further fixed three negative, not statistically significant residual variances to zero; and correlated some residual variances. Except for the student sub-sample, model fit indices were around the recommended thresholds.

**Table 8:** **Loadings, residual variances, and reliability of the scale-based Alberta Context Tool sub-scales**.

		**Care aides (*****N*** = 251**)**			**Nurses (*****N*** = 179**)**			**Allied (*****N*** = 141**)**			**Managers (*****N*** = 118**)**			**Students (*****N*** = 58**)**			**All (N=752)**		
**Factor**	**Item**	**λ**	**δ**	**ω (α)**	**λ**	**δ**	**ω (α)**	**λ**	**δ**	**ω (α)**	**λ**	**δ**	**ω (α)**	**Λ**	**δ**	**ω (α)**	**λ**	**δ**	**ω (α)**
LEAD	L1	0.620	0.535		0.740	0.564		0.584	0.740		0.638	0.464		0.517	0.614		0.649	0.574	
	L2	0.396	0.850		0.415	0.889		0.653	0.674		0.468	0.896		0.305	1.087		0.459	0.865	
	L3	0.609	0.703		0.722	0.690		0.595	0.527		0.647	0.654		0.581	0.670		0.648	0.661	
	L4	0.709	0.253		0.735	0.414		0.651	0.305		0.712	0.305		0.699	0.200		0.709	0.310	
	L5	0.765	0.371		0.988	0.343		0.906	0.254		0.797	0.275		0.920	0.089		0.867	0.311	
	L6	0.726	0.326	0.828 (0.846)	0.852	0.400	0.857 (0.851)	0.759	0.322	0.859 (0.857)	0.734	0.279	0.848 (0.837)	0.728	0.441	0.819 (0.782)	0.774	0.360	0.845 (0.845)
CULT	C1	0.463	0.523		0.750	0.413		0.548	0.584		0.513	0.475		0.646	0.410		0.587	0.494	
	C4	0.547	0.629		0.408	0.821		0.449	0.837		0.550	0.644		0.627	0.509		0.510	0.711	
	C5	0.361	0.412		0.278	0.363		0.231	0.379		0.224	0.471		0.194	0.267		0.292	0.394	
	C6	0.632	0.268	0.687 (0.674)	0.625	0.412	0.679 (0.674)	0.374	0.275	0.553 (0.547)	0.301	0.470	0.550 (0.506)	0.453	0.210	0.725 (0.715)	0.534	0.349	0.655 (0.644)
FEED	F1	1.061	0.364		0.966	0.566		1.027	0.257		0.701	1.040		1.168	0.402		1.097	0.569	
	F2	1.093	0.325		0.939	0.474		1.047	0.280		0.950	0.691		1.151	0.268		1.107	0.421	
	F3	1.034	0.265		0.979	0.286		1.010	0.089		0.995	0.586		1.094	0.240		1.083	0.294	
	F4	1.096	0.234		1.056	0.251		1.091	0.048		1.137	0.303		1.042	0.114		1.147	0.212	
	F5	1.186	0.052		1.155	0.055		1.096	0.009		1.202	0.119		1.158	0.116		1.227	0.061	
	F6	0.903	0.569	0.957 (0.966)	0.851	0.574	0.941 (0.958)	0.828	0.340	0.973 (0.976)	0.710	1.074	0.895 (0.914)	0.881	0.568	0.961 (0.965)	0.903	0.621	0.952 (0.961)
SCAP	SC1	0.464	0.352		0.341	0.291		0.506	0.310		0.389	0.359		0.425	0.226		0.443	0.311	
	SC2	0.599	0.351		0.367	0.459		0.492	0.500		0.376	0.376		0.488	0.406		0.527	0.382	
	SC4	0.492	0.314		0.441	0.463		0.277	0.579		0.352	0.436		0.476	0.318		0.446	0.415	
	SC5	0.590	0.254		0.528	0.248		0.590	0.244		0.380	0.190		0.344	0.193		0.517	0.255	
	SC6	0.697	0.332	0.834 (0.825)	0.655	0.377	0.747 (0.755)	0.682	0.409	0.761 (0.782)	0.605	0.471	0.707 (0.736)	0.608	0.290	0.793 (0.779)	0.646	0.404	0.790 (0.787)
STAF	ST1	0.749	0.786		0.895	0.722		0.780	0.530		1.170	0.155		0.728	0.705		0.904	0.604	
	ST2	1.080	0.000	0.810 (0.771)	0.849	0.0506	0.712 (0.704)	0.944	0.373	0.767 (0.770)	0.944	0.608	0.854 (0.852)	.957	0.222	0.754 (0.752)	0.951	0.374	0.779 (0.776)
SPAC	SP2	.933	0.289		0.783	0.522		0.745	0.381		1.073	0.000		1.009	0.155		0.929	0.281	
	SP3	1.358	0.615	0.853 (0.829)	1.626	0.000	0.917 (0.797)	1.276	0.569	0.811 (0.763)	1.050	1.010	0.817 (0.798)	1.407	0.608	0.884 (0.863)	1.317	0.729	0.833 (0.808)
TIME	T1	0.551	0.753		0.610	0.369		0.505	0.648		0.807	0.334		0.459	0.452		0.591	0.643	
	T2	0.684	0.417		0.681	0.366		0.648	0.737		0.670	0.720		0.449	0.309		0.753	0.454	
	T3	0.754	0.304		0.611	0.484		0.671	0.644		0.715	0.620		0.635	0.295		0.692	0.483	
	T4	0.836	0.505	0.801 (0.801)	0.740	0.564	0.805 (0.820)	0.584	0.740	0.735 (0.728)	0.638	0.464	0.802 (0.840)	0.517	0.614	0.790 (0.785)	0.794	0.511	0.793 (0.794)

*λ, factor loadings (all p < 0.000); δ, residual variances; ω, reliability based on factor model parameters; α, Cronbach's Alpha; LEAD, Leadership; CULT, Culture; FEED, Feedback; SCAP, Social Capital; STAF, OS Staffing; SPAC, OS Space; TIME, OS Time*.

Fit of ACT model 3a (three count-based concepts) was poor. This model could not be estimated in the allied group. Our analysis of the model and the item contents indicated that the factors Informal Interactions and Resources should be split into two and three factors, respectively. In this model we also removed two ill-fitting items (Informal Interactions item 11, informal bedside teaching sessions, and resources item 11, in-services in your facility) and correlated residual variances (see Figure 2, model 3b). In addition, we allowed cross-loading of one item. Except for the allied and managers sub-sample, model fit indices were around the recommended thresholds.

A 13-factor model (combination of models 2b and 3b) fit poorly and could not be estimated reliably in the sub-samples of managers and student due to a non-positive first-order derivative product matrix.

#### Factor structure of the conceptual research utilization scale

Fit of the CRU Scale model (Figure 2, model 4) was excellent. In this model we allowed the residual variances of items 1 and 2 to correlate in all groups and the residual variances of items 4 and 5 in the managers group.

#### Reliability

Using the item loadings and residual variances of these final models (2b, 3b, and 4), we calculated the reliability (ω) of each ACT sub-scale and the CRU Scale (Tables 8–**10**). Except for Culture, all scale-based ACT concepts were acceptably reliable (ω > 0.7) in all provider groups (Table 8). The same is true for the count-based ACT concepts of Informal Interactions with direct care providers (IINT_a_), Reading Resources (RESO_a_), and Electronic Resources (RESO_c_) (Table 9). Reliability of instructive/informative resources (RESO_b_) only falls below.7 in the student group. Less reliable ACT concepts are Formal Interactions (FINT; ω > 0.7 only in the nurses group and in the entire sample), and Informal Interactions with indirect care providers (IINT_b_; ω > 0.7 only in the student group). Reliability of the CRU Scale is excellent in all groups (Table 10).

**Table 9:** **Loadings, residual variances, and reliability of the count-based Alberta Context Tool sub-scales**.

		**Care aides (*****N*** = 263**)**			**Nurses (*****N*** = 187**)**			**Allied (*****N*** = 147**)**			**Managers (*****N*** = 117**)**			**Students (*****N*** = 63**)**			**All (*****N*** = 777**)**		
**Factor**	**Item**	**λ**	**δ**	**ω (α)**	**λ**	**δ**	**ω (α)**	**λ**	**δ**	**ω (α)**	**λ**	**δ**	**ω (α)**	**λ**	**δ**	**ω (α)**	**λ**	**δ**	**ω (α)**
FINT	FI1	0.789	0.527		0.596	0.484		0.788	0.781		0.493	0.964		0.665	0.590		0.682	0.706	
	FI2	0.737	0.227		0.833	0.559		0.832	0.116		0.849	0.737		0.823	0.180		0.920	0.348	
	FI5	0.489	0.628	0.746 (0.727)	0.405	0.990	0.623 (0.610)	0.356	1.041	0.668 (0.622)	0.523	0.863	0.576 (0.526)	0.424	1.184	0.652 (0.656)	0.545	0.897	0.703 (0.683)
IINT_a_	II1	0.740	0.450		0.561	0.337		0.740	0.881		0.923	0.047		0.846	0.282		0.662	0.671	
	II2	0.681	0.494		0.506	0.536		0.869	0.596		0.544	0.194		0.691	0.497		0.639	0.617	
	II4	0.790	0.828		0.604	0.796		0.901	0.717		0.675	0.763		0.769	0.553		0.655	0.956	
	II5	—	—		—	—		—	—		0.205	0.878		—	—		—	—	
	II10	1.030	0.586	0.817 (0.777)	0.999	0.840	0.740 (0.654)	0.965	0.715	0.806 (0.800)	0.464	1.025	0.731 (0.736)	1.141	0.341	0.877 (0.830)	1.035	0.588	0.760 (0.750)
IINT_b_	II4	0.415	0.828		0.306	0.796		—	—		0.288	0.763		0.363	0.553		0.326	0.956	
	II5	0.735	0.318		0.617	0.542		0.373	0.409		0.344	0.878		0.780	0.242		0.549	0.561	
	II6	0.626	0.230	0.696 (0.597)	0.800	0.358	0.636 (0.540)	0.838	0.277	0.681 (0.524)	0.908	0.619	0.512 (0.441)	0.776	0.314	0.769 (0.711)	0.831	0.327	0.612 (0.569)
RESO_a_	R1	0.222	0.153		0.419	0.377		0.399	0.660		0.708	0.740		0.326	0.265		0.471	0.411	
	R2	0.622	0.293		0.745	0.416		0.897	0.683		0.776	0.635		0.825	0.599		0.795	0.510	
	R3	0.776	0.376	0.761 (0.686)	0.824	0.381	0.771 (0.733)	0.820	0.445	0.715 (0.684)	1.096	0.348	0.794 (0.812)	0.871	0.183	0.796 (0.771)	0.913	0.409	0.781 (0.758)
RESO_b_	R4	0.538	1.362		0.621	1.347		0.680	1.270		0.428	1.475		0.773	0.889		0.547	1.394	
	R5	1.282	0.357		0.848	0.680		1.073	1.175		1.004	0.391		0.566	1.281		1.158	0.575	
	R6	1.105	0.452	0.798 (0.753)	0.816	0.783	0.650 (0.713)	1.377	0.512	0.768 (0.733)	0.999	0.516	0.713 (0.669)	0.950	0.839	0.635 (0.719)	1.215	0.468	0.778 (0.733)
RESO_c_	R7	1.217	0.204		1.290	1.140		1.376	1.335		1.225	0.730		1.112	1.231		1.413	1.073	
	R8	0.945	0.287		0.885	1.191		0.784	2.038		1.101	1.800		0.670	1.593		0.968	1.359	
	R9	0.508	0.411		1.225	0.358		1.320	0.653		1.471	0.758		0.634	0.444		1.337	0.450	
	R10	0.277	0.218	0.886 (0.798)	0.946	0.466	0.857 (0.874)	1.230	.356	0.835 (0.863)	1.253	0.946	0.858 (0.860)	0.633	0.579	0.707 (0.822)	1.094	0.479	0.873 (0.892)

*λ, factor loadings (all p < 0.000); δ, residual variances; ω, reliability based on factor model parameters; α, Cronbach's Alpha; FINT, Formal Interactions; IINT_a–b_, Informal Interactions factors a and b; RESO_a–c_, Resources factors a, b, and c*.

**Table 10:** **Loadings, residual variances, and reliability of the CRU Scale**.

		**Care aides (*N* = 268)**			**Nurses (*N* = 183)**			**Allied (*N* = 147)**			**Managers (*N* = 123)**			**Students (*N* =63)**			**All (*N* = 790)**		
**Factor**	**Item**	**λ**	**δ**	**ω (α)**	**λ**	**δ**	**ω (α)**	**λ**	**δ**	**ω (α)**	**λ**	**δ**	**ω (α)**	**λ**	**δ**	**ω (α)**	**λ**	**δ**	**ω (α)**
CORU	CRU1	1.076	0.403		0.897	0.447		1.093	0.375		1.053	0.387		0.670	0.340		1.074	0.409	
	CRU2	1.154	0.220		0.907	0.398		1.180	0.229		1.144	0.218		0.660	0.288		1.133	0.282	
	CRU3	1.187	0.184		1.064	0.297		1.132	0.362		1.134	0.120		0.829	0.335		1.181	0.255	
	CRU4	1.129	0.196		1.056	0.292		1.233	0.168		1.149	0.360		0.786	0.233		1.154	0.239	
	CRU5	1.146	0.230	0.963 (0.966)	1.111	0.140	0.942 (0.946)	1.240	0.331	0.959 (0.961)	1.111	0.249	0.959 (0.962)	0.683	0.321	0.897 (0.903)	1.186	0.222	0.959 (0.962)

*λ, factor loadings (all p < 0.000); δ, residual variances; ω, reliability based on factor model parameters; α, Cronbach's Alpha; CORU, Conceptual Research Utilization*.

### Measurement invariance

Results of the measurement invariance analyses are summarized in Table 11. The ACT scale-based model (2b) demonstrated partial weak measurement invariance after we freed three (2.1%) of the 145 fixed loadings (29 in each of the five provider group models). After freeing eight (5.5%) of the 145 item intercepts this model also demonstrated partial strong measurement invariance. However, we found neither strict nor partially strict measurement invariance for this model.

**Table 11:** **Results of the measurement invariance analyses**.

**Model**	**Invariance**	**χ^2^**	**df**	***p***	**RMSEA (90%CI)**	**CFI**	**TLI**	**SRMR**	**χ^2^diff**	**dfdiff**	**pdiff**
ACT, Scale-Based	Configural	1998.108	1746	0.0000	0.031 (0.024-0.037)	0.947	0.938	0.064	—		—
	Weak	2123.086	1835	0.0000	0.032 (0.025-0.039)	0.939	0.933	0.075	169.854	89	0.0000
	Partial Weak^a^	2087.54	1833	0.0000	0.030 (0.023-0.037)	0.946	0.941	0.069	102.468	87	0.1231
	Strong	2256.038	1921	0.0000	0.034 (0.028-0.040)	0.930	0.926	0.074	299.021	88	0.0000
	Partial Strong^b^	2171.729	1913	0.0000	0.030 (0.023-0.036)	0.946	0.942	0.071	99.212	80	0.7160
	Strict	247.612	2048	0.0000	0.037 (0.031-0.042)	0.911	0.912	0.074	36.091	135	0.0000
ACT, Count-Based	Configural	804.527	667	0.0002	0.036 (0.026-0.045)	0.952	0.939	0.062	—	—	—
	Weak	994.059	722	0.0000	0.049 (0.042-0.057)	0.905	0.888	0.089	239.697	55	0.0000
	Partial Weak^c^	857.915	714	0.0002	0.036 (0.026-0.045)	0.950	0.940	0.068	61.745	47	0.0730
	Strong	1071.458	766	0.0000	0.051 (0.043-0.058)	0.894	0.881	0.085	359.936	52	0.0000
	Partial Strong^d^	899.960	753	0.0002	0.035 (0.025-0.044)	0.949	0.942	0.069	49.057	39	0.1298
	Strict	1297.781	829	0.0000	0.060 (0.054-0.067)	0.837	0.832	0.115	489.513	76	0.0000
CRU Scale	Configural	15.286	19	0.7042	0.000 (0.000-0.054)	1.000	1.004	0.008	—	—	—
	Weak	34.011	35	0.5157	0.000 (0.000-0.055)	1.000	1.001	0.043	26.271	16	0.0503
	Strong	58.041	51	0.2318	0.030 (0.000-0.061)	0.997	0.997	0.051	31.482	16	0.0117
	Partial Strong^e^	53.366	50	0.3462	0.021 (0.000-0.057)	0.999	0.999	0.049	23.714	15	0.0701
	Strict	94.710	74	0.0527	0.042 (0.000-0.066)	0.991	0.994	0.046	41.155	24	0.0160
	Partial Strict^f^	85.957	73	0.1425	0.034 (0.000-0.059)	0.994	0.996	0.049	32.919	23	0.0825

a *Loadings of three items freed: C1 (care aides), ST2 and SP3 (Managers)*.

b *Intercepts of eight items freed: T1 and T3 (care aides), T3 (nurses), SP2 and T1 (allied providers), F1 (managers), F4 and T3 (students)*.

c *Loadings of eight items freed: II6, R9, and R10 (care aides), II2 (allied providers), II1, II10, and R1 (managers), R5 (students)*.

d *Intercepts of 13 items freed: FI1, FI5, R3, R4, and R9 (care aides), II10 and R3 (nurses), II1, II4, II5, and II10 (allied providers), II1 and R3 (managers)*.

e *Intercept of one item freed: CRU4 (managers)*.

f *Residual variance of one item freed: CRU2 (nurses); df, degrees of freedom; RMSEA, Root Mean Square Error of Approximation; 90%CI, 90% Confidence Interval; CFI, Comparative Fit Index; TLI, Tucker Lewis Index; SRMR, Standardized Root Mean Square Error*.

The count-based ACT model (3b) also demonstrated partial weak measurement invariance after we freed eight (8.2%) of the 98 fixed loadings. Partial strong measurement invariance was met after we freed 13 (13.7%) of the 95 fixed item intercepts. As for the model of the ACT scale-based items (2b), we were not able to obtain strict or partially strict measurement invariance for this model.

The CRU Scale proved to be fully weak invariant as well as partially strong invariant (only one [4%] of the 25 fixed item intercepts was freed). Additionally, after freeing one (4%) of the fixed residual variances, this Scale showed partial strict measurement invariance.

## Discussion

Mounting evidence links organizational context in health care to care provider outcomes of quality of work life, attitudes toward research utilization, and use of best practice, and to patient outcomes of safety and quality of care (Glisson, 2002; Greenhalgh et al., 2004; Meijers et al., 2006; Kaplan et al., 2010; Aarons et al., 2012; Grabowski et al., 2014; Harvey et al., 2015). However, we lack robust quantitative studies that use sound theory and validated research tools to assess and better understand organizational context factors and how they affect these outcomes (Kaplan et al., 2010; Flodgren et al., 2012). This is particularly true in the LTC sector (Boström et al., 2012). Robust research tools are a prerequisite for such studies (Graham et al., 2010; Proctor et al., 2011; Proctor and Brownson, 2012), but only a few robust tools are available to assess organizational context and research utilization in LTC settings (Squires et al., 2011b; Chaudoir et al., 2013).

The German versions of the ACT and the CRU Scale are to date the only tools available to assess modifiable factors of organizational context and the extent of conceptual research utilization in German LTC facilities. Applying *The Standards*, we generated evidence for the validity of scores derived from these tools based on feedback from content experts and the tool developers (instrument content evidence; Hoben et al., 2013), and based on responses from target persons (response process evidence; Hoben et al., 2014a). This paper presents the assessment of the third source of validity evidence: the internal structure of the instruments. We demonstrated that both the scale-based ACT concepts and the CRU Scale reflect the hypothesized seven- and one-factor structure, respectively. This corresponds with the findings of the Canadian validation studies (Estabrooks et al., 2011b; Squires et al., 2011a, 2015). The finding of an ill-fitting three-factor model of the count-based ACT concepts also is in agreement with the Canadian results (Estabrooks et al., 2011b; Squires et al., 2015).

We removed four items from the scale-based ACT model, which negatively influenced the model fit. care aides in particular frequently asked for the meaning of the Culture item 2 (are you a member of a supportive work group) during data collection. Culture item 3 (organization effectively balances best practice and productivity; Hoben et al., 2014a). Social Capital item 3 does not focus on the unit level (asks for the exchange with other teams in the facility), and therefore clearly differs from the five other Social Capital items. Space item 1 (private space available on this unit or floor) was also removed in the analysis of the Canadian tool (Estabrooks et al., 2011b), and in the German study participants frequently struggled with the meaning of this item.

In the count-based ACT model, we also removed two items. Informal bedside teaching sessions (Informal Interactions item 11) are not very common in German nursing homes. In contrast, in-services (Resources item 11) are very common. Almost all continuing education happens inside the facilities, and care providers rarely are sent to trainings outside their facility. In both cases, the result is almost no variance in the item responses.

In contrast to the Canadian validation studies (Estabrooks et al., 2011b; Squires et al., 2015), we not only assessed the factor structure of the ACT care aides version (separate models for care aides and nursing students) and nurses, but also of the allied and manager versions. In order to compare the factor models, we not only excluded items contributing to model ill-fit, but also items not included in all ACT versions. Furthermore, we decided to split up the Informal Interactions factor and the Resources factor in two, and three factors, respectively. Informal interactions take place with persons providing direct care to residents (care aides, nurses, allied providers) and, with indirect care providers (e.g., quality improvement specialists, clinical educators), reflecting two somewhat distinct concepts. The allied group includes both direct care providers (e.g., recreational therapists) and indirect care providers (e.g., the social service leader). Therefore, the item referring to interactions with allied providers is associated with both Informal Interaction factors in all groups except the allied group itself. Resources items refer to three types of resources: reading resources (e.g., a library, text books, journals), instructive/informative resources (e.g., notice boards, policies/procedures, clinical practice guidelines), and electronic resources (e.g., computer connected to the internet, software assisting with care and decision making, electronic reminders, websites on the internet). This reflects the factor structure of the count-based ACT concepts that was found by Estabrooks et al. (Estabrooks et al., 2009b) using a principal component analysis.

Our factor models did not fit perfectly. χ^2^ tests of most models were statistically significant. Relevance of the χ^2^ test in relation to relative model fit indices for model fit evaluation has been debated at length. The most radical position is to ban relative model fit indices completely (Barrett, 2007) and to only accept p values of χ^2^ tests that are above. 75 (Hayduk, 1996; Hayduk and Glaser, 2000; Hayduk et al., 2007). The χ^2^ test is the only available statistical test for the null hypothesis that the sample covariance matrix (data collected) matches the model-implied covariance matrix. Its p value is the probability of observing the sample covariance matrix if the model-implied covariance matrix reflected the true population values (Hayduk, 1996; Hayduk and Glaser, 2000; Hayduk et al., 2007). However, the ACT items do not reflect truly redundant indicators of the concepts under which they are subsumed, therefore we did not expect that our factor models would be perfectly well-specified. We assessed model fit using recognized relative fit indices and looking for local areas of strain. When meeting these criteria, even a model with a significant χ^2^ value can be accepted as a reasonably close approximation to reality (Bentler, 2000; Millsap, 2007; Mulaik, 2007).

This paper adds two unique insights to the literature on the validity of the two tools: information on measurement invariance across provider groups and reliability based on latent variable model parameters. The ACT models showed partial strong measurement invariance (>80% of the loadings and intercepts restricted to be equal across the groups), indicating that the constructs to be measured (Leadership, Culture, Feedback, etc.) are the same in all groups and are measured equally across groups (Byrne et al., 1989; Brown, 2006; Dimitrov, 2010; Sass, 2011; Wang and Wang, 2012). The CRU Scale showed partial strict measurement invariance (>80% of the residual variances restricted to be equal across groups). This means, that not only the construct is the same across groups and is measured equally across groups, but that the amount of unexplained variance (including measurement error) also is equal across groups. It is not surprising that the ACT models were not strictly measurement invariant. The provider groups differ substantially in various characteristics (age, language skills, qualification, job experience, education), influencing their ability to understand the questionnaire instructions and items. Therefore, we expected the residual variances to differ substantially between provider groups. These results indicate that comparisons between the provider groups should not be based on the observed items or item scores, but rather on models accounting for the different residual variances (latent variable models; Byrne et al., 1989; Brown, 2006; Dimitrov, 2010; Sass, 2011; Wang and Wang, 2012).

### Limitations

Although we reached an appropriate sample size (821 responses from 38 facilities) and although we applied a strong sampling method (stratified random sampling), some sampling limitations must be noted. Many facilities refused to participate in the study, mainly due to staffing problems. Therefore, the included facilities likely represent a selection of facilities with more favorable organizational context. We assume that facilities agreeing to participate were motivated, rated themselves as well organized, and felt they had sufficient resources to participate.

The optimal methods to determine the required sample size for CFA are model-based methods, such as the procedure described by Satorra and Saris (1985), and the approach of Muthén and Muthén (2002) based on Monte Carlo simulations. However, they require information on model parameters based on previous studies, information that was not available. Our overall sample size exceeded requirements based on common rules of thumb discussed in the literature, such as a minimum sample size of 100–200 cases, 5–10 cases per observed variable, or 5–10 cases per parameter to be estimated in the model (Brown, 2006; Kline, 2011b; Lee et al., 2012; Wang and Wang, 2012; Hancock and Mueller, 2013). However, our student and manager sub-samples were rather small (n = 65 and n = 129, respectively), given the relatively complex ACT factor models. Further studies could determine sample size requirements more precisely by using parameter estimates and effect sizes from this study to inform model-based approaches.

Post-hoc modification of latent variable models based solely on modification indices is problematic, as with each modification the covariance residuals become more and more biased, their distribution does not correspond to a χ^2^ distribution any longer, and the risk of type I error (accepting a failing model) increases (capitalization by chance) (MacCallum, 1986; MacCallum et al., 1992). As recommended by Silvia and MacCallum (Silvia and MacCallum, 1988) we therefore based our model modifications not primarily on the modification indices but rather on theory (item contents, experiences during data collection, discussions with instrument developers, and logic). However, in this study we were not able to test our final models in an independent sample, as our sample size was not large enough to randomly split the sample in two halves. Future studies should therefore further test these models in independent samples.

## Conclusions

The scores of the German versions of the ACT and the CRU Scale are sufficiently reliable and valid. Building on two previous publications providing validity evidence based on instrument contents and response processes, this study provides validity evidence based on the internal structure of the tools. The ACT items reflect seven scale-based concepts and six count-based concepts of organizational context. The CRU Scale items reflect one common factor. These findings supplement the results of the Canadian validation studies, indicating that robust translation and adaptation methods can retain the good psychometric properties of original tools. This study extends the international findings on the psychometric properties of the ACT and the CRU Scale by adding information on their measurement invariance across different provider groups. Partial strong measurement invariance (ACT) and partial strict measurement invariance (CRU Scale) indicate that the concepts are measured equally well across the different provider groups. However, as the ACT lacked strict measurement invariance, observed variables (or scale scores based on observed variables) cannot be compared between provider groups. Instead, group comparisons should be based on latent variable models, which take into account the different residual variances of each group. The two translated tools will facilitate robust research on organizational context factors and their association with research utilization in German LTC facilities – an important prerequisite to improving quality of care and quality of life of LTC residents.

## Author contributions

MH conducted the data collections, statistical analyses, and prepared figures, tables and a first draft of this manuscript. CE and JS are the developers of the Alberta Context Tool and the Research Utilization Scale. They gave methodological input, reviewed the analyses and drafts, and critically reviewed and revised the manuscript. JB was MH's primary PhD supervisor and critically reviewed and revised the manuscript.

## Funding

MH conducted this study as part of his PhD work. MH was member of an interdisciplinary Graduate Program on Dementia, located at the Network Aging Research (NAR), University of Heidelberg, Germany, and received a Doctoral Fellowship funded by the Robert Bosch Foundation, Stuttgart, Germany. The funder had no role in study design, data collection and analysis, decision to publish, or preparation of the manuscript.

### Conflict of interest statement

The authors declare that the research was conducted in the absence of any commercial or financial relationships that could be construed as a potential conflict of interest.
