# Supplementary material for: Factor Structure, Reliability and Measurement Invariance of the Alberta Context Tool and the Conceptual Research Utilization Scale, for German Residential Long Term Care
Source: Front Psychol. 2016 Sep 7;7:1339. doi: 10.3389/fpsyg.2016.01339 (PMC5013130; doi:10.3389/fpsyg.2016.01339)
Supplement: Supplementary file 1 [file Table1.docx]

Supplementary Material

Factor structure, reliability and measurement invariance of the Alberta Context Tool and the Conceptual Research Utilization Scale, for German residential long term care

Matthias Hoben*, Carole A. Estabrooks, Janet E. Squires, Johann Behrens

***Correspondence:** mhoben@ualberta.ca

## Supplementary Table 1. Rationale for the correlation of residual variances in the ACT and CRU scale factor models

| **Provider group** | **Modell** | **Correlated residual variances** | **Justification (common source of “error”)** |
| --- | --- | --- | --- |
| **Care aides** | ACT 2b | L1 with L2 | L1 asks if the leader seeks for feedback even if it is difficult to hear. L2 asks if the leader focusses on success rather than failures. For both items HCAs often provided us with the feedback, they never had thought about those questions and did not know exactly what to answer. |
|  | ACT 3b | II1 with II2 | II1 asks for interactions with HCAs, II2 asks for interactions with RNs. Direct care providers (HCAs, RNs, AHPs, and students), in particular, frequently appeared to not read carefully enough and not differentiate sufficiently between these two items. |
|  |  | II8 with II10 | II8 asks for interactions with persons who bring new ideas to the unit, II10 asks for informal interactions in general. HCAs frequently seemed to think of the same persons in both cases. |
| **Nurses** | ACT 2b | SC1 with SC2 | SC1 asks if team members exchange information, SC2 asks if observations of resident conditions are regularly taken seriously by persons in positions of authority. RNs and managers (many of them RNs with some additional education) linked those two issues closely. |
|  |  | T2 with T3 | T2 asks how often the participant has time to look something up in the literature, T3 asks how often participants have time to talk to someone about resident care. RNs and managers (many of them RNs with some additional education) stated that those things are closely interrelated. |
|  | ACT 3b | II1 with II2 | II1 asks for interactions with HCAs, II2 asks for interactions with RNs. Direct care providers (HCAs, RNs, AHPs, and students), in particular, frequently appeared to not read carefully enough and not differentiate sufficiently between these two items. |
|  |  | R5 with R6 | R5 asks for policies and procedures, R6 asks for clinical practice guidelines. RNs and students, in particular, found it difficult sometimes to differentiate between these two types of resources. |
|  |  | R7 with R8 | R7 asks for a computer connected to the internet, R8 asks for computer programs to assist with care and decision making. For direct care providers who frequently document their care tasks (RNs, AHPs, and students), in particular, the use of a computer during their work was identical to these documentation tasks (and thus to using the respective software). |
| **Allied** | ACT 2b | SC2 with SC4 | SC2 asks if observations of resident conditions are regularly taken seriously by persons in positions of authority, SC4 asks if the participant feels comfortable talking about resident care issues to persons in positions of authority. In the AHP group these persons were often identical. |
|  | ACT 3b | II1 with II2 | II1 asks for interactions with HCAs, II2 asks for interactions with RNs. Direct care providers (HCAs, RNs, AHPs, and students), in particular, frequently appeared to not read carefully enough and not differentiate sufficiently between these two items. |
|  |  | R7 with R8 | R7 asks for a computer connected to the internet, R8 asks for computer programs to assist with care and decision making. For direct care providers who frequently document their care tasks (RNs, AHPs, and students), in particular, the use of a computer during their work was identical to these documentation tasks (and thus to using the respective software). |
| **Managers** | ACT 2b | SC1 with SC2 | SC1 asks if team members exchange information, SC2 asks if observations of resident conditions are regularly taken seriously by persons in positions of authority. RNs and managers (many of them RNs with some additional education) linked those two issues closely. |
|  |  | T2 with T3 | T2 asks how often the participant has time to look something up in the literature, T3 asks how often participants have time to talk to someone about resident care. RNs and managers (many of them RNs with some additional education) stated that those things closely interrelated. |
|  | ACT 3b | FI1 with FI5 | FI1 asks for team meetings about residents, FI5 asks for continuing education held outside of the facility. In both cases the leaders frequently thought of themselves as the persons who organized those events, not as participants. |
|  |  | II2 with II10 | II2 asks for informal interactions with RNs, II10 asks for informal interactions in general. RNs were the persons with whom leaders most frequently interacted. |
|  |  | R1 with R2 | R1 asks for the use of textbooks, R2 asks for the use of a library. Frequently the leaders reported their own pool of textbooks as the ‘library’ in the facility. |
|  | CRU 4 | CRU4 with CRU5 | CRU4 asks how often new research knowledge has led to new ideas on how to care for residents, CRU5 asks how often new research knowledge helped participants make sense of things they have been doing for residents. Leaders frequently did not know the difference between these questions. |
| **Students** | ACT 2b | C5 with C6 | C5 asks if participants think that people in their facility work to provide what residents need, C6 asks if participants are members of a supportive work group. For students those two issues were closely related or even viewed as identical. |
|  | ACT 3b | II1 with II2 | II1 asks for interactions with HCAs, II2 asks for interactions with RNs. Direct care providers (HCAs, RNs, AHPs, and students), in particular, frequently appeared to not read carefully enough and not differentiate sufficiently between these two items. |
|  |  | R5 with R6 | R5 asks for policies and procedures, R6 asks for clinical practice guidelines. Especially RNs and students found it difficult sometimes to differentiate between these two types of resources. |
|  |  | R7 with R8 | R7 asks for a computer connected to the internet, R8 asks for computer programs to assist with care and decision making. For direct care providers who frequently document their care tasks (RNs, AHPs, and students), in particular, the use of a computer during their work was identical to these documentation tasks (and thus to using the respective software). |
| **Whole sample** | ACT3b | II1 with II2 | II1 asks for interactions with HCAs, II2 asks for interactions with RNs. Direct care providers (HCAs, RNs, AHPs, and students), in particular, frequently appeared to not read carefully enough and not differentiate sufficiently between these two items. |
|  |  | R5 with R6 | R5 asks for policies and procedures, R6 asks for clinical practice guidelines. RNs and students, in particular, found it difficult sometimes to differentiate between these two types of resources. |
| **All samples** | ACT 2b | F1-F4 (all possible correlations) | In the German version these items contain the word statistics, which some participants did not understand or found confusing in this context. |
|  | CRU 4 | CRU1 with CRU2 | CRU1 asks how often research knowledge gave participants new ideas on how to care for residents, CRU2 asks how research knowledge raised their awareness about new ways to care for residents. Both items refer to new ways of caring for residents. |

ACT: Alberta Context Tool; CRU: Conceptual Research Utilization; L: Leadership; C: Culture; FI: Formal Interactions; II: Informal Interactions; R: Resources; SC: Social Capital; T: Time
